# Supplementary material for: Patient and clinician perceptions of telehealth in musculoskeletal physiotherapy services - A systematic review of the evidence-base
Source: PLOS Digit Health. 2025 Mar 31;4(3):e0000789. doi: 10.1371/journal.pdig.0000789 (PMC11957330; doi:10.1371/journal.pdig.0000789)
Supplement: S1 Panel — (DOCX) [file pdig.0000789.s002.docx]

***Table demonstrating PEO Search strategy Terms.***

|  | **P – Population** | | **E - Exposure** | | **0 - Outcome** | |  |
| --- | --- | --- | --- | --- | --- | --- | --- |
| **Keywords** | **Musculoskeletal Physiotherapy** | AND | **Telehealth** | AND | **Patient/clinician Satisfaction** | NOT |  |
| **MeSH Terms** | OR |  | OR |  | OR |  |  |
| AMED | (SU.EXACT("PHYSICAL THERAPY SPECIALITY") OR SU.EXACT("PHYSICAL MEDICINE") OR SU.EXACT("PHYSICAL THERAPY MODALITIES") OR SU.EXACT("SPORTS MEDICINE") OR SU.EXACT("EXERCISE THERAPY") OR SU.EXACT("REHABILITATION SPECIALITY")) | AND  AND | (SU.EXACT("TELECOMMUNICATIONS") OR SU.EXACT("TELEMEDICINE") OR SU.EXACT("TELEPHONE")) | AND  AND | (SU.EXACT("JOB SATISFACTION") OR SU.EXACT("PERSONAL SATISFACTION") OR SU.EXACT("PATIENT COMPLIANCE") OR SU.EXACT("PATIENT SATISFACTION") OR SU.EXACT("PATIENT ACCEPTANCE OF HEALTH CARE") OR SU.EXACT("CONSUMER SATISFACTION")) | NOT  NOT | N/A |
| EMACRE | PHYSIOTHERAPY/ OR PHYSICAL MEDICINE/ OR PHYSIOTHERAPY PRACTICE/ |  | TELEHEALTH/ OR TELEMEDICINE/ |  | JOB SATISFACTION/ OR SATISFACTION/ OR CUSTOMER SATISFACTION/ OR PATIENT SATISFACTION/ OR LIFE SATISFACTION/ |  |  |
| EMBASE | HOME PHYSIOTHERAPY/ OR PHYSIOTHERAPY PRACTICE/ OR PHYSIOTHERAPY/ |  | TELEHEALTH/ OR TELEMEDICINE/ OR TELECONSULTATION/ OR TELEDIAGNOSIS/ OR TELEREHABILITATION/ OR TELETHERAPY/ OR VIDEO CONSULTATION/ |  | EXP JOB SATISFACTION/ OR EXP PATIENT SATISFACTION/ OR LIFE SATISFACTION/ OR JOB SATISFACTION ASSESSMENT/ OR EXP SATISFACTION/ |  |  |
| PsycINFO  MEDLINE  CINAHL | (MH "PHYSICAL THERAPY SPECIALTY") OR (MH "PHYSICAL THERAPISTS") OR (MH "PHYSICAL THERAPY MODALITIES") OR "PHYSIOTHERAPY" OR (MH "PHYSICAL THERAPIST ASSISTANTS") OR (MH "REHABILITATION") (MH "THE CHARTERED SOCIETY OF PHYSIOTHERAPY") OR (MH "AUSTRALIAN PHYSIOTHERAPY ASSOCIATION") OR (MH "PHYSIOTHERAPY EVIDENCE DATABASE") OR (MH "CANADIAN PHYSIOTHERAPY ASSOCIATION") OR (MH "STUDENTS, PHYSICAL THERAPY") OR (MH "PHYSICAL THERAPY PRACTICE, RESEARCH-BASED") OR (MH "PHYSICAL THERAPY PRACTICE, EVIDENCE-BASED") OR (MH "WORLD CONFEDERATION FOR PHYSICAL THERAPY") DE "REHABILITATION" OR DE "EXERCISE THERAPY" OR DE "PHYSICAL THERAPY" |  | (MH "TELEMEDICINE") OR (MH "REMOTE CONSULTATION") OR (MH "TELEPATHOLOGY") OR (MH "DIGITAL HEALTH") OR (MH "TELEREHABILITATION") (MH "TELEHEALTH") OR (MH "TELECOMMUNICATIONS") OR (MH "TELECONFERENCING") OR (MH "TELEMEDICINE") OR (MH "VIDEOCONFERENCING") (DE "TELEMEDICINE" OR DE "TELEPHONE SYSTEMS" OR DE "TELEREHABILITATION") OR (DE "ONLINE THERAPY") |  | (MH "PATIENT SATISFACTION") OR (MH "PERSONAL SATISFACTION") OR (MH "JOB SATISFACTION") OR (MH "PATIENT COMPLIANCE") OR (MH "ATTITUDE TO HEALTH") OR (MH "TREATMENT ADHERENCE AND COMPLIANCE") OR (MH "PATIENT ACCEPTANCE OF HEALTH CARE") OR (MH "PATIENT PREFERENCE") (MH "JOB SATISFACTION") OR (MH "PERSONAL SATISFACTION") OR (MH "PATIENT SATISFACTION") OR (MH "CONSUMER SATISFACTION") OR (MH "ATTITUDE") DE "SATISFACTION" OR DE "CLIENT SATISFACTION" OR DE "CONSUMER SATISFACTION" OR DE "JOB SATISFACTION" OR DE "ROLE SATISFACTION" OR DE "CONTENTMENT" OR DE "DISSATISFACTION" OR DE "PHYSICAL COMFORT" |  |  |
|  | OR |  | OR |  | OR |  |  |
| **Synonyms** | (Physiotherapy)  (Physiotherapist*)  (Physical Therap*)  (Physio Therap*)  (Musculoskeletal System*)  (Physical treatment*)  (Rehabilitation)  (Recreational Therap*)  (Physical Medicine)  (Movement Therap*)  (Activity Therap*)  (Exercise Therap*)  (Mobility Therap*)  (Ambulation Therap*) | AND | (Tele-health)  (Telemedicine)  (Tele-medicine)  (Mobile Health)  (mHealth)  (m-health)  (eHealth)  (e-health)  (Remote Consultation*)  (Teleconsultation*)  (Tele-consultation*)  (Telepathology)  (Telerehabilitation*)  (Tele-rehabilitation*)  (Virtual Rehabilitation*)  (Teleradiology)  (Telecommunication)  (Telegraphy)  (Telegraphies)  (Teleconference*)  (Videoconferencing)  (Telediagnosis)  (e-rehabilitation)  (Remote rehabilitation)  (Tele-rehab)  (Telerehab)  (Telephysiotherapy)  (Tele-Physiotherapy) | AND | (Satisfaction)  (Preference*)  (Behavior*)  (Fulfilment)  (Contentment)  (Well-being)  (Outcome*)  (Evaluation*)  (Effectiveness)  (Efficac*)  (Success*)  (Perception*)  (Compliance)  (seeking)  (Attitude*)  (Qualit*)  (Relationship*)  (Inclination*)  (Disposition*)  (Proprietary)  (Ownership) | NOT | (Mental)  (Anxiet*)  (Depression)  (Psych*7)  (Addiction) OR  (Violence)  (Neuropsycholog*)  (Weight Loss)  (Obesity)  (Nutrition)  (Neurolog*)  (Nervous system*)  (Neuromuscular)  (Stroke)  (Brain Injury)  (Cognitive)  (Parkinson*)  (Neuro)  (Neurorehabilitation)  (Sclerosis)  (Dementia)  (Women* health)  (Obstetric*)  (Gynaecolog*)  (Gynecolog*)  (Incontinence)  (Pelvi*)  (Pulmonary)  (Lung)  (Asthma)  (Respiratory)  (Bronchiectasis)  (Speech)  (Tinnitus)  (Vision)  (Hearing)  (Implant)  (Cancer*)  (Neoplasm*)  (Palliative)  (Transplant*)  (Myocardial)  (Coronary)  (Haemophilia*)  (Hemophillia*)  (Dermatolog*)  (Diabet*)  (Endocrine)  (Burn*)  (Rheumatology) |
