# Supplementary material for: Patient and clinician perceptions of telehealth in musculoskeletal physiotherapy services - A systematic review of the evidence-base
Source: PLOS Digit Health. 2025 Mar 31;4(3):e0000789. doi: 10.1371/journal.pdig.0000789 (PMC11957330; doi:10.1371/journal.pdig.0000789)
Supplement: S2 Panel — (DOCX) [file pdig.0000789.s003.docx]

**Supporting file 3** Search strategy and results.

**Databases searched:** CINAHL (EBSCO), EMBASE (OVID), EMCARE (OVID), PsycINFO (EBSCO), Medline (EBSCO), AMED (ProQuest)

**Database:** Medline, CINAHL, PsycINFO (EBSCO)

**Date:** 13^th^ August 2024

**No. of results:** 194

| # | Search | Number |
| --- | --- | --- |
| S1 – Medline | (MH "Physical Therapy Specialty") OR (MH "Physical Therapists") OR (MH "Physical Therapy Modalities") OR "Physiotherapy" OR (MH "Physical Therapist Assistants") OR (MH "Rehabilitation") | 219,251 |
| S2 - CINAHL | (MH "The Chartered Society of Physiotherapy") OR (MH "Australian Physiotherapy Association") OR (MH "Physiotherapy Evidence Database") OR (MH "Canadian Physiotherapy Association") OR (MH "Students, Physical Therapy") OR (MH "Physical Therapy Practice, Research-Based") OR (MH "Physical Therapy Practice, Evidence-Based") OR (MH "World Confederation for Physical Therapy") | 10,385 |
| S3 - PsycINFO | DE "Rehabilitation" OR DE "Exercise Therapy" OR DE "Physical Therapy" | 164,997 |
| S4 | S1 OR S2 OR S3 | 310,369 |
| S5 – Medline | (MH "Telemedicine") OR (MH "Remote Consultation") OR (MH "Telepathology") OR (MH "Digital Health") OR (MH "Telerehabilitation") | 72,927 |
| S6 - CINAHL | (MH "Telehealth") OR (MH "Telecommunications") OR (MH "Teleconferencing") OR (MH "Telemedicine") OR (MH "Videoconferencing") | 88,847 |
| S7 - PsycINFO | (DE "Telemedicine" OR DE "Telephone Systems" OR DE "Telerehabilitation") OR (DE "Online Therapy") | 72,126 |
| S8 | S5 OR S6 OR S7 | 128,443 |
| S9 - Medline | (MH "Patient Satisfaction") OR (MH "Personal Satisfaction") OR (MH "Job Satisfaction") OR (MH "Patient Compliance") OR (MH "Attitude to Health") OR (MH "Treatment Adherence and Compliance") OR (MH "Patient Acceptance of Health Care") OR (MH "Patient Preference") | 535,506 |
| S10 - CINAHL | (MH "Job Satisfaction") OR (MH "Personal Satisfaction") OR (MH "Patient Satisfaction") OR (MH "Consumer Satisfaction") OR (MH "Attitude") | 333,033 |
| S11 - PsycINFO | DE "Satisfaction" OR DE "Client Satisfaction" OR DE "Consumer Satisfaction" OR DE "Job Satisfaction" OR DE "Role Satisfaction" OR DE "Contentment" OR DE "Dissatisfaction" OR DE "Physical Comfort" | 131,699 |
| S12 | S9 OR S10 OR S11 | 678,081 |
| S13 – Medline, CINAHL, PsycINFO | (Physiotherapy) OR (Physiotherapist*) OR (Physical Therap*) OR (Physio Therap*) OR (Musculoskeletal System*) OR (Physical treatment*) OR (Rehabilitation) OR (Recreational Therap*) OR (Physical Medicine) OR (Movement Therap*) OR (Activity Therap*) OR (Exercise Therap*) OR (Mobility Therap*) OR (Ambulation Therap*) | 1,561,146 |
| S14 - Medline, CINAHL, PsycINFO | (Tele-health) OR (Telemedicine) OR (Tele-medicine) OR (Mobile Health) OR (mHealth) OR (m-health) OR (eHealth) OR (e-health) OR (Remote Consultation*) OR (Teleconsultation*) OR (Tele-consultation*) OR (Telepathology) OR (Telerehabilitation*) OR (Tele-rehabilitation*) OR (Virtual Rehabilitation*) OR (Teleradiology) OR (Telecommunication) OR (Telegraphy) OR (Telegraphies) OR (Teleconference*) OR (Videoconferencing) OR (Telediagnosis) OR (e-rehabilitation) OR (Remote rehabilitation) OR (Tele-rehab) OR (Telerehab) OR (Telephysiotherapy) OR (Tele-Physiotherapy) | 216,423 |
| S15 - Medline, CINAHL, PsycINFO | (Satisfaction) OR (Preference*) OR (Behavior*) OR (Fulfilment) OR (Contentment) OR (Well-being) OR (Outcome*) OR (Evaluation*) OR (Effectiveness) OR (Efficac*) OR (Success*) OR (Perception*) OR (Compliance) OR (seeking) OR (Attitude*) OR (Qualit*) OR (Relationship*) OR (Inclination*) OR (Disposition*) OR (Proprietary) OR (Ownership) | 20,688,185 |
| S16 | S4 OR S13 | 1,561,146 |
| S17 | S8 OR S14 | 230,541 |
| S18 | S12 OR S15 | 20,700,576 |
| S19 | S16 AND S17 AND S18 | 18,712 |
| S20 | S16 AND S17 AND S18  Limiters - Publication Date: 20190101-20241231; Peer Reviewed; English language; Age Related: All Adult: 19+ years; Language: English Expanders - Apply equivalent subjects Search modes - Proximity | 6,762 |
| S21 | (Mental) OR (Anxiet*) OR (Depression) OR (Psych*7) OR (Addiction) OR (Violence) OR (Neuropsycholog*) OR (Weight Loss) OR (Obesity) OR (Nutrition) OR (Neurolog*) OR (Nervous system*) OR (Neuromuscular) OR (Stroke) OR (Brain Injury) OR (Cognitive) OR (Parkinson*) OR (Neuro) OR (Neurorehabilitation) OR (Sclerosis) OR (Dementia) OR (Women* health) OR (Obstetric*) OR (Gynaecolog*) OR (Gynecolog*) OR (Incontinence) OR (Pelvi*) OR (Pulmonary) OR (Lung) OR (Asthma) OR (Respiratory) OR (Bronchiectasis) OR (Speech) OR (Tinnitus) OR (Vision) OR (Hearing) OR (Implant) OR (Cancer*) OR (Neoplasm*) OR (Palliative) OR (Transplant*) OR (Myocardial) OR (Coronary) OR (Haemophilia*) OR (Hemophillia*) OR (Dermatolog*) OR (Diabet*) OR (Endocrine) OR (Burn*) OR (Rheumatology) OR (Cardiac) OR (Cardio) OR (Child*) OR (Paed*) OR (paediatric*) OR (Adolesen*) OR (Infant*) OR (Neonate*) | 2,651,532 |
| S22 | S20 NOT S21 | 529 |
| S23 | S22  Limiters - Linked Full Text; Publication Date: 20190101-20241231; Peer Reviewed; English language; PDF Full Text; Age Related: All Adult: 19+ years; Publication Type: Journal Article; Language: English; Publication Year: 2019-2024; Publication Type: Peer Reviewed Journal; Language: English; Age Groups: Adulthood (18 yrs & older); Document Type: Journal Article; Exclude Dissertations; Exclude MEDLINE records; Language: English; Publication Type: Academic Journal; Age Groups: All Adult Expanders - Apply equivalent subjects Search modes - Proximity | 194 |

**Database:** AMED (ProQuest)

**Date:** 13^th^ August 2024

**No. of results:** 104

| # | Searches | Number |
| --- | --- | --- |
| S1 | (SU.EXACT("PHYSICAL THERAPY SPECIALITY") OR SU.EXACT("PHYSICAL MEDICINE") OR SU.EXACT("PHYSICAL THERAPY MODALITIES") OR SU.EXACT("SPORTS MEDICINE") OR SU.EXACT("EXERCISE THERAPY") OR SU.EXACT("REHABILITATION SPECIALITY")) | 21713 |
| S2 | (SU.EXACT("TELECOMMUNICATIONS") OR SU.EXACT("TELEMEDICINE") OR SU.EXACT("TELEPHONE")) | 1691 |
| S3 | (SU.EXACT("JOB SATISFACTION") OR SU.EXACT("PERSONAL SATISFACTION") OR SU.EXACT("PATIENT COMPLIANCE") OR SU.EXACT("PATIENT SATISFACTION") OR SU.EXACT("PATIENT ACCEPTANCE OF HEALTH CARE") OR SU.EXACT("CONSUMER SATISFACTION")) | 6670 |
| S4 | ((Physiotherapy) OR (Physiotherapist*) OR (Physical Therap*) OR (Physio Therap*) OR (Musculoskeletal System*) OR (Physical treatment*) OR (Rehabilitation) OR (Recreational Therap*) OR (Physical Medicine) OR (Movement Therap*) OR (Activity Therap*) OR (Exercise Therap*) OR (Mobility Therap*) OR (Ambulation Therap*)) | 125780 |
| S5 | ((Tele-health) OR (Telemedicine) OR (Tele-medicine) OR (Mobile Health) OR (mHealth) OR (m-health) OR (eHealth) OR (e-health) OR (Remote Consultation*) OR (Teleconsultation*) OR (Tele-consultation*) OR (Telepathology) OR (Telerehabilitation*) OR (Telerehabilitation*) OR (Virtual Rehabilitation*) OR (Teleradiology) OR (Telecommunication) OR (Telegraphy) OR (Telegraphies) OR (Teleconference*) OR (Videoconferencing) OR (Telediagnosis) OR (e-rehabilitation) OR (Remote rehabilitation) OR (Tele-rehab) OR (Telerehab) OR (Telephysiotherapy) OR (Tele-Physiotherapy)) | 2527 |
| S6 | ((Satisfaction) OR (Preference*) OR (Behavior*) OR (Fulfilment) OR (Contentment) OR (Well-being) OR (Outcome*) OR (Evaluation*) OR (Effectiveness) OR (Efficac*) OR (Success*) OR (Perception*) OR (Compliance) OR (seeking) OR (Attitude*) OR (Qualit*) OR (Relationship*) OR (Inclination*) OR (Disposition*) OR (Proprietary) OR (Ownership)) | 144759 |
| S7 | S4 OR S1 | 125962 |
| S8 | S5 OR S2 | 2794 |
| S9 | S6 OR S3 | 145346 |
| S10 | S9 AND S8 AND S7 | 1245 |
| S11 | (S10 NOT ((Mental) OR (Anxiet*) OR (Depression) OR (Psych*7) OR (Addiction) OR (Violence) OR (Neuropsycholog*) OR (Weight Loss) OR (Obesity) OR (Nutrition) OR (Neurolog*) OR (Nervous system*) OR (Neuromuscular) OR (Stroke) OR (Brain Injury) OR (Cognitive) OR (Parkinson*) OR (Neuro) OR (Neurorehabilitation) OR (Sclerosis) OR (Dementia) OR (Women* health) OR (Obstetric*) OR (Gynaecolog*) OR (Gynecolog*) OR (Incontinence) OR (Pelvi*) OR (Pulmonary) OR (Lung) OR (Asthma) OR (Respiratory) OR (Bronchiectasis) OR (Speech) OR (Tinnitus) OR (Vision) OR (Hearing) OR (Implant) OR (Cancer*) OR (Neoplasm*) OR (Palliative) OR (Transplant*) OR (Myocardial) OR (Coronary) OR (Haemophilia*) OR (Hemophillia*) OR (Dermatolog*) OR (Diabet*) OR (Endocrine) OR (Burn*) OR (Rheumatology) OR (Cardiac) OR (Cardio) OR (Child*) OR (Paed*) OR (paediatric*) OR (Adolesen*) OR (Infant*) OR (Neonate*))) and (pd(20190101-20241231) and ud(20190101-20241231)) and (abany(yes)) and (la.exact("English")) | 104 |

**Database:** EMBASE (OVID)

**Date:** 1st August 2024

**No. of results:** 20

| **#** | **Searches** | **Number** |
| --- | --- | --- |
| **S1** | physiotherapy/ or physical medicine/ or physiotherapy practice/ | 120492 |
| **S2** | telehealth/ or telemedicine/ | 68815 |
| **S3** | job satisfaction/ or satisfaction/ or customer satisfaction/ or patient satisfaction/ or life satisfaction/ | 305602 |
| **S4** | (Physiotherapy or Physiotherapist* or Physical Therap* or Physio Therap* or Musculoskeletal System* or Physical treatment* or Rehabilitation or Recreational Therap* or Physical Medicine or Movement Therap* or Activity Therap* or Exercise Therap* or Mobility Therap* or Ambulation Therap*).mp. [mp=title, abstract, heading word, drug trade name, original title, device manufacturer, drug manufacturer, device trade name, keyword heading word, floating subheading word, candidate term word] | 660809 |
| **S5** | (Tele-health or Telemedicine or Tele-medicine or Mobile Health or mHealth or mhealth or eHealth or e-health or Remote Consultation* or Teleconsultation* or Teleconsultation* or Telepathology or Telerehabilitation* or Tele-rehabilitation* or Virtual Rehabilitation* or Teleradiology or Telecommunication or Telegraphy or Telegraphies or Teleconference* or Videoconferencing or Telediagnosis or e-rehabilitation or Remote rehabilitation or Tele-rehab or Telerehab or Telephysiotherapy or TelePhysiotherapy).mp. [mp=title, abstract, heading word, drug trade name, original title, device manufacturer, drug manufacturer, device trade name, keyword heading word, floating subheading word, candidate term word] | 138937 |
| **S6** | (Satisfaction or Preference* or Behavior* or Fulfilment or Contentment or Well-being or Outcome* or Evaluation* or Effectiveness or Efficac* or Success* or Perception* or Compliance or seeking or Attitude* or Qualit* or Relationship* or Inclination* or Disposition* or Proprietary or Ownership).mp. [mp=title, abstract, heading word, drug trade name, original title, device manufacturer, drug manufacturer, device trade name, keyword heading word, floating subheading word, candidate term word] | 15821156 |
| S7 | S1 OR S4 | 660809 |
| S8 | S2 OR S5 | 149924 |
| S9 | S3 OR S6 | 15821156 |
| S10 | S7 AND S8 AND S9 | 8278 |
| S11 | limit 10 to (ovid full text available and human and english language and embase and yr="2019 - 2024" and (adult or aged )) | 20 |

**Database:** EMCARE (OVID)

**Date:** 2nd August 2024

**No. of results:** 76

| **#** | **Searches** | **Number** |
| --- | --- | --- |
| S1 | home physiotherapy/ or physiotherapy practice/ or physiotherapy/ | 37,157 |
| S2 | telehealth/ or telemedicine/ or teleconsultation/ or telediagnosis/ or telerehabilitation/ or teletherapy/ or video consultation/ | 25,705 |
| S3 | exp job satisfaction/ or exp patient satisfaction/ or life satisfaction/ or job satisfaction assessment/ or exp satisfaction/ | 114,542 |
| S4 | (Physiotherapy or Physiotherapist* or Physical Therap* or Physio Therap* or Musculoskeletal System* or Physical treatment* or Rehabilitation or Recreational Therap* or Physical Medicine or Movement Therap* or Activity Therap* or Exercise Therap* or Mobility Therap* or Ambulation Therap*).mp. [mp=title, abstract, heading word, drug trade name, original title, device manufacturer, drug manufacturer, device trade name, keyword heading word] | 215,081 |
| **S5** | (Tele-health or Telemedicine or Tele-medicine or Mobile Health or mHealth or m-health or eHealth or e-health or Remote Consultation* or Teleconsultation* or Tele-consultation* or Telepathology or Telerehabilitation* or Tele-rehabilitation* or Virtual Rehabilitation* or Teleradiology or Telecommunication or Telegraphy or Telegraphies or Teleconference* or Videoconferencing or Telediagnosis or e-rehabilitation or Remote rehabilitation or Tele-rehab or Telerehab or Telephysiotherapy or Tele-Physiotherapy).mp. [mp=title, abstract, heading word, drug trade name, original title, device manufacturer, drug manufacturer, device trade name, keyword heading word] | 53,785 |
| **S6** | (Satisfaction or Preference* or Behavior* or Fulfilment or Contentment or Well-being or Outcome* or Evaluation* or Effectiveness or Efficac* or Success* or Perception* or Compliance or seeking or Attitude* or Qualit* or Relationship* or Inclination* or Disposition* or Proprietary or Ownership).mp. [mp=title, abstract, heading word, drug trade name, original title, device manufacturer, drug manufacturer, device trade name, keyword heading word] | 4,036,723 |
| S7 | S1 OR S4 | 215,081 |
| S8 | S2 OR S5 | 58,198 |
| S9 | S3 OR S6 | 4,036,723 |
| S10 | S7 AND S8 AND S9 | 2,869 |
| S11 | limit 10 to (full text and human and english language and yr="2019 - 2024" and (adult <18 to 64 years> or aged <65+ years>)) | 76 |
