# Supplementary material for: Patient and clinician perceptions of telehealth in musculoskeletal physiotherapy services - A systematic review of the evidence-base
Source: PLOS Digit Health. 2025 Mar 31;4(3):e0000789. doi: 10.1371/journal.pdig.0000789 (PMC11957330; doi:10.1371/journal.pdig.0000789)
Supplement: S1 Table — (DOCX) [file pdig.0000789.s004.docx]

**Supporting file 4** List of excluded studies

| **Authors** | **Year** | **Title** | **Journal** | **Reason for exclusion** |
| --- | --- | --- | --- | --- |
| R. Allen-McHugh, A. Stephens and A. Tyree  M. Elhadi, A. Msherghi, A. Elhadi, A. Ashini, A. Alsoufi, F. Bin Alshiteewi, et al.  A. M. Dennett, N. F. Taylor, K. Williams, A. K. Lewis, P. Brann, J. D. Hope, et al.  M. Szekeres and K. Valdes  J. S. Lee, A. Bhatt, L. M. Pollack, S. L. Jackson, J. E. Chang, X. Tong, et al.  N. S. Cox, K. Scrivener, A. E. Holland, L. Jolliffe, A. Wighton, S. Nelson, et al.  Z. D. Rethorn, A. C. Lee and T. J. Rethorn  M. Wakasa, T. Odashima, A. Saito, M. Kimoto, I. Saito, S. Handa, et al.  S. Weissman, A. Gladin and T. E. Davenport  M. H. Ross, M. Nelson, V. Parravicini, M. Weight, R. Tyrrell, N. Hartley, et al. | 2022  2021  2022  2022  2024  2021  2021  2020  2023  2023 | Rural Early Intervention Therapists’ Perceptions of Emergency Remote Telehealth Use  Utilization of Telehealth Services in Libya in Response to the COVID-19 Pandemic: Cross-sectional Analysis  Consumer perspectives of telehealth in ambulatory care in an Australian health network  Virtual health care & telehealth: Current therapy practice patterns  Telehealth use during the early COVID-19 public health emergency and subsequent health care costs and utilization  A brief intervention to support implementation of telerehabilitation by community rehabilitation services during COVID-19: a feasibility study  Connecting at the webside: rapid telehealth implementation for musculoskeletal clinicians  Telerehabilitation with tablet computers replaces face-to-face rehabilitation  Using clinical decision making to identify the appropriateness of telehealth physical therapy: a case series  Staff perspectives on the key elements to successful rapid uptake of telerehabilitation in medium‐sized public hospital physiotherapy departments | Archives of Physical Medicine and Rehabilitation 2022 Vol. 103 Issue 12 Pages e162-e16  JMIR Med Inform 2021 Vol. 9 Issue 2 Pages e23335  Health Soc Care Community 2022 Vol. 30 Issue 5 Pages 1903-1912  Journal of Hand Therapy 2022 Vol. 35 Issue 1 Pages 124-130  Health affairs scholar 2024 Vol. 2 Issue 1 Pages qxae001  Archives of Physical Medicine and Rehabilitation 2021 Vol. 102 Issue 4 Pages 789-795  Journal of Orthopaedic & Sports Physical Therapy 2021 Vol. 51 Issue 1 Pages 8-11  Physical & Occupational Therapy in Geriatrics 2020 Vol. 38 Issue 1 Pages 85-97  Orthopaedic Physical Therapy Practice 2023 Vol. 35 Issue 1 Pages 31  Physiotherapy Research International 2023 Vol. 28 Issue 3 Pages e1991 | Poster Presentation  Incorrect participants  Incorrect participants  Incorrect participants  Incorrect participants  Incorrcet outcome  Incorrect outcome  Incorrect outcome  Incorrect outcome  Incorrect outcome |
