# Supplementary material for: Patient and clinician perceptions of telehealth in musculoskeletal physiotherapy services - A systematic review of the evidence-base
Source: PLOS Digit Health. 2025 Mar 31;4(3):e0000789. doi: 10.1371/journal.pdig.0000789 (PMC11957330; doi:10.1371/journal.pdig.0000789)
Supplement: Attachment — Submitted filename: Response_to_reviewer_letter_PLOS Final.docx [file pdig.0000789.s006.docx]

31.12.2024

Dear Reviewer,

Thank you for your consideration and helpful comments on this manuscript. Please find below responses to each of the comments explaining where and which changes have been made.

**Journal Requirements:**

1. In the online submission form, you indicated that "All relevant data are within the manuscript and its Supporting Information files.".

All PLOS journals now require all data underlying the findings described in their manuscript to be freely available to other researchers, either:

1. In a public repository,

2. Within the manuscript itself, or

3. Uploaded as supplementary information.

This policy applies to all data except where public deposition would breach compliance with the protocol approved by your research ethics board. If your data cannot be made publicly available for ethical or legal reasons (e.g., public availability would compromise patient privacy), please explain your reasons by return email and your exemption request will be escalated to the editor for approval. Your exemption request will be handled independently and will not hold up the peer review process but will need to be resolved should your manuscript be accepted for publication. One of the Editorial team will then be in touch if there are any issues.

***All relevant data has been included within the supporting information section including search strategy and results, a list of excluded studies with reasons for exclusion, and a PRISMA checklist.***

2. We have noticed that you have uploaded Supporting Information files, but you have not included a list of legends. Please add a full list of legends for your Supporting Information files after the references list.

***A full list of legends has been added within the ‘Supporting Information’ section of the manuscript.***

3. We notice that your supplementary files are uploaded with the file type 'Manuscript'. Please amend the file type to 'Supporting Information'. Please ensure that each Supporting Information file has a legend listed in the manuscript after the references list.

***This has been amended to supporting information.***

4. As required by our policy on Data Availability, please ensure your manuscript or supplementary information includes the following:

A numbered table of all studies identified in the literature search, including those that were excluded from the analyses.

***The search strategy and results has been included within the supporting information section (S3 File. Search strategy and results).***

***A list of excluded studies has been included within the supporting information section (S4 File. List of excluded studies) and can also be viewed within the PRISMA flow chart (Fig 1. Prisma flow chart) and within the narrative (Selection of articles)***.

For every excluded study, the table should list the reason(s) for exclusion.

***The reasons for study exclusion can be found within the PRISMA flowchart and within supporting information section (S4 File. List of excluded studies).***

If any of the included studies are unpublished, include a link (URL) to the primary source or detailed information about how the content can be accessed.

***All the included studies are published. Please see line 193 within the manuscript for amendments.***

A table of all data extracted from the primary research sources for the systematic review and/or meta-analysis. The table must include the following information for each study:

Name of data extractors and date of data extraction

***Name and date of data extractors has been added, please see line 194.***

Confirmation that the study was eligible to be included in the review.

***This information is provided within Fig 1. Prisma flow chart.***

All data extracted from each study for the reported systematic review and/or meta-analysis that would be needed to replicate your analyses.

***This information is provided within Table 2. – Characteristics of studies.***

If data or supporting information were obtained from another source (e.g. correspondence with the author of the original research article), please provide the source of data and dates on which the data/information were obtained by your research group.

***Not applicable.***

If applicable for your analysis, a table showing the completed risk of bias and quality/certainty assessments for each study or outcome. Please ensure this is provided for each domain or parameter assessed. For example, if you used the Cochrane risk-of-bias tool for randomized trials, provide answers to each of the signalling questions for each study. If you used GRADE to assess certainty of evidence, provide judgements about each of the quality of evidence factor. This should be provided for each outcome.

***Please see addition of risk of bias tables 3 – 6 these have been included within the manuscript but note that the option to include these in a supporting information file may be preferred.***

An explanation of how missing data were handled.

***All the included studies were published and available for review.***

Reviewer #1: the paper has fundamental shortcomings and lacks quality writing, and innovation. However, recommendations for improvement have been provided. Best wishes

Abstract:

•Clarify the specific types of musculoskeletal conditions addressed in the reviewed studies.

***This systematic review gives an overview of telehealth within musculoskeletal services. Please see changes to title and abstract (line 14).***

•Provide more precise details on the databases searched and date ranges.

***Please see correction to abstract (line 15/16).***

•Include the specific number of studies reviewed after screening, not just the initial search results.

***Please see correction to abstract (line 17).***

•Mention any limitations or gaps identified in the current literature on this topic.

***Please see correction to abstract (line 25 - 31).***

•Strengthen the conclusion by highlighting specific implications for practice and future research directions.

***Please see correction to abstract (line 25 - 31).***

Methods:

•Provide more details on the search strategy, including the full list of search terms and how they were combined.

***Search terms, search strategy and results can be found in the supporting information***.

Discussion:

•Elaborate on the implications of these findings for clinical practice, health policy, and future research.

***Corrections have been made with specific reference to implications for clinical practice, health policy and future research. Please see amendments within the discussion and future recommendations sections.***

•Address the limitations of the review more comprehensively, including potential biases in the included studies and in the review process itself.

***Corrections have been made to the strengths and limitations section.***

•Provide more specific recommendations for future research, identifying key areas where knowledge gaps remain.

***More specific recommendations for future research have been made including types of studies to address long term outcomes and view of satisfaction with telehealth within musculoskeletal condition specific care.***

Reviewer #2: - Add in the research objectives and research questions as in SLR in Introduction

***This has been moved to the end of introduction section, please see corrections to this section.***

- Add in the reviewer background (AS) and (SI) such how many years’ experience

***Please see corrections to the strengths and limitations section which acknowledges the contributions of the authors and their extensive clinical and academic background.***

- Highlight in figure 1 - other source from where?

***Please see corrections to the PRISMA flow chart. Citation and reference list searching has been added to other sources.***

Reviewer #3: The authors examined the impact of telehealth on patient and clinician satisfaction within physiotherapy and provided insightful recommendations to improve future quality of research in this field. However, this work still contains some inadequacies in the analyses and discussions. Although the authors vigorously reviewed previous studies and summarized them, they only characterized the findings to date. They need to add more specific analyses or discussion to the current manuscript. I listed some issues that need to be addressed to improve this work mature enough for publication in PLOS Digital Health.

1.Although the authors present the characteristics of the studies in Table2, the clinical profiles of patients such as osteoarthritis, rheumatoid arthritis, cerebrovascular disease, spinal canal stenosis, etc. are lacking. If the studies the authors referred to include information on patient profiles, they should conduct a deeper analysis of the positive and negative themes of telehealth based on the patients’ profiles.

***The primary aim of study is to understand satisfaction of telehealth within musculoskeletal physiotherapy services from both patient and clinician perspectives. Addition of the term ‘services’ throughout the paper has hopefully improved clarity. It was felt that research that investigated condition specific satisfaction was too broad and beyond the scope of this paper to answer the research aims and objectives. However, recommendations have been made that future research should look to address this as this will help with implications for clinical practice such a telehealth integration, adopting patient-centred telehealth, and expanding regulatory and policy considerations (See future recommendations section).***

2.In the “3. Flexibility” subsection of the Result section, the authors explored flexibility as a positive theme of telehealth. However, it is unclear what the flexibility in telehealth means. The authors need to provide a clear definition of flexibility in telehealth.

***Corrections have been made to clarify the meaning of ‘flexibility’ within the studies. This denotes flexibility in terms of healthcare delivery between remote and in-person care allowing for better patient choice. (Please see amendments line 250-253).***

3.What types of physiotherapy tend to be accepted as telehealth, and what types tend not to be accepted? The authors need to explore the details of physiotherapy menu and provide the tendency in the “7. Lack of Physical Contact” subsection. In addition, the authors should discuss more specifically the possibility of a hybrid approach in terms of the type of physiotherapy.

***The primary aim of study is to understand satisfaction of telehealth within musculoskeletal physiotherapy services from both patient and clinician perspectives. It was felt that research that investigated the spectrum of physiotherapy professionalisms (musculoskeletal, cardiorespiratory, geriatric, paediatrics etc.) was too broad and beyond the scope of this paper to answer the research aims and objectives.***

***The hybrid approach to telehealth has been discussed with amendments within the discussion section. This specifically relates to hybrid approach within musculoskeletal physiotherapy as this aligns to the research aims and objectives.***

4.If the authors intend to discuss the future integration of telehealth within routine clinical practice, they should be more specific about how each negative theme could be addressed. For example, with the lack of physical contact, are there digital technologies that can solve this problem? Could virtual or augmented reality (VR or AR) as telerehabilitation improve the negative perception of the lack of physical contact? The authors should discuss the potential of emerging digital technology to address the current problems.

***Amendments have been made which acknowledge the role advanced technologies within telehealth and how future studies may depict differing view of telehealth satisfaction in terms of negative themes identified. (See amendments to line 461 – 462).***

Reviewer #4: Overall, I think this is an excellent review. The discussion is very well done. I really liked the recognition of a paradoxical benefit/harm with technological literacy and cost of travel for patients. The suggestion to have future studies investigating perception after the pandemic vs. intra pandemic is great, was hoping this would be discussed. See below for comments and suggestions.

Would be clearer on physiotherapy - I believe the authors are primarily talking about physical therapy the discipline but would at least clarify that there are other disciplines (occupational and speech therapy) that are often performed in concert and linked. Are these less amenable or more amenable to telerehabilitation services? Is there a reason why these disciplines were not included in this review?

***The primary aim of study is to understand satisfaction of telehealth within musculoskeletal physiotherapy services from both patient and clinician perspectives. It was felt that research that investigated the multiple allied health professions was too broad and beyond the scope of this paper to answer the research aims and objectives.***

I think the lack of physical contact should be more discussed. This is a significant limitation in the ability to provide appropriate and corrective physiotherapy. If you are unable to be hands on and training appropriate movement, bad habits can be entrained, which can result in injuries and lack of progress.

***Amendments have been made within the discussion that reflects the decision-making processes required to appropriately determine when remote or in-person care is needed. This has been reflected as an implication for clinical practice with recommendations for future policy and regulation at a local level.***

Does having physiotherapy as part of primary care make sense? Efficient use of those services frequently involves physiatrists (PM&R) providing a MSK diagnosis or focus for physiotherapists to target.

***The author acknowledges the comments made by the reviewer and appreciates this may reflect a contrasting delivery of health care within musculoskeletal services globally. Within the UK, physiotherapy is an integral part of primary care, and many physiotherapists work autonomously in diagnosis and management or as part of a self-referral system. This study aims to support the work of physiotherapists already working within this sector and to aid in research that may impact clinical practice and future research.***

Many thanks to the reviewers and editorial team for the extensive and detailed feedback that have enhanced this paper. We look forward to your future correspondence regarding this study.

Yours Sincerely,

Anthony Smith (Postgraduate Musculoskeletal Lecturer)

and

Dr Sue Innes (Postgraduate Musculoskeletal and Sonography Lead)

apsmitb@essex.ac.uk

inness@essex.ac.uk
